# Mental Time Travel and Time Reference Difficulties in Alzheimer’s Disease: Are They Related? A Systematic Review

**DOI:** 10.3389/fpsyg.2022.858001

**Published:** 2022-05-09

**Authors:** Evodie Schaffner, Mélanie Sandoz, Cristina Grisot, Noémie Auclair-Ouellet, Marion Fossard

**Affiliations:** ^1^Faculté des Lettres et Sciences Humaines, Institut des Sciences Logopédiques, University of Neuchâtel, Neuchâtel, Switzerland; ^2^Zurich Center for Linguistics, University of Zurich, Zurich, Switzerland; ^3^Social Research and Demonstration Corporation, Ottawa, ON, Canada

**Keywords:** time conceptualization, mental time travel, time reference, Alzheimer’s disease, verbal inflection

## Abstract

Mental time travel and language enable us to go back and forth in time and to organize and express our personal experiences through time reference. People with Alzheimer’s disease have both mental time travel and time reference impairments, which can greatly impact their daily communication. Currently, little is known about the potential relationship between time conceptualization (i.e., mental time travel) and time reference difficulties in this disease. A systematic review of the literature was performed to determine if this link had already been investigated. Only three articles integrated both time conceptualization and time reference measures. However, the link between the two was not systematically analyzed and interpreted. This review highlights the lack of research addressing the question of the influence of time conceptualization impairments in Alzheimer’s disease on other cognitive domains, and especially language.

## Introduction

Progressive loss in episodic memory is a risk factor for cognitive decline and dementia and a core criterion for Alzheimer’s disease (AD; Albert et al., 2011; McKhann et al., 2011). According to Tulving (2002), episodic memory refers to the memory of past and personal experiences that are spatially and temporally anchored (see also Renoult and Rugg, 2020 for a theoretical review). It allows us to re-experience past events with vivid details but also to project ourselves into future events (Schacter et al., 2007). This capacity to experience the passage of time and to navigate in time, also known as mental time travel, contributes to a sense of identity and self-continuity (Conway, 2005). Consequently, besides experiencing difficulties in recollecting past events, the decrease in episodic memory abilities in AD leads to other disorders. It is currently well-documented that people with AD may experience disruptions in the sense of self (El Haj et al., 2019), anxiety regarding future events (Kloszewska, 1998), and deficits in time conceptualization (e.g., interval judgment and recall of past events; Requena-Komuro et al., 2020).

Concerning the latter, several studies have shown how time conceptualization is characteristically affected in AD, using various tasks focusing on different time intervals. For instance, some of these studies assessed the ability to make temporal judgements about short intervals (i.e., seconds) and highlighted that AD participants show time distortions (i.e., overestimation or underestimation of time intervals), compared to healthy, aged participants (e.g., Carrasco et al., 2000; Papagano et al., 2004; El Haj et al., 2013). Other tasks focused on longer intervals (i.e., days, months, or years) and showed that people with AD experience difficulties navigating between different periods of life (i.e., difficulties apprehending the present or imagining the future and interferences of the past in the present; Shiromaru-Sugimoto et al., 2018). The Autobiographical Interview (AI; Levine et al., 2002) has been widely used to assess the ability to travel mentally in time through discourse production. In this task, AD participants must recall and recount personal events from different periods (their youth, their middle age, and their recent past), and imagine future events (Addis and Tippett, 2004; Addis et al., 2009; Irish et al., 2011, 2012a,b). Based on participant narratives, two categories of discursive details are generally extracted: internal and external detail. Details that are directly linked to the event are classified as *internal details*. They convey information about the time, place, or emotions that are related to the event and rely heavily on episodic memory processes. In contrast, *external details* provide semantic information that is not directly linked to the event (e.g., general semantic knowledge, metacognitive statements, etc.) and involve semantic memory processes specifically. These studies showed that AD participants have specific difficulties reliving past events and using past experiences to project themselves in the future, and that those difficulties are related to episodic memory impairment. Indeed, compared to control participants, they produce less internal details for each period, suggesting an impairment in mental time travel, while no such difference is observed for external details.

The combination of internal and external details in a narrative builds the temporal framework of the reported event, but other language markers such as verbal inflection and temporal adverbs can also be used to express the temporality of the event in a more precise way and testify to mental time travel. Language through time reference (i.e., the linguistic expression of time) is indeed an important way to express concepts of past, present, and future that are related to every human experience (Benveniste, 1974; Harner, 1982; Zhang and Hudson, 2018). From a linguistic perspective, time reference concerns three main categories: tense and grammatical aspect (which are grammatical categories), and lexical aspect (a lexical category). These categories are essential to organize the localization of events on the timeline (Grisot, 2018), indicating when and how the events took place. While tense refers to *when* the event took place (i.e., before, at the same time, or after the time of speech, referring, respectively, to a past, present, or future event; Reichenbach, 1947), aspect indicates *how* the event took place. More precisely, grammatical aspect corresponds to the degree of completion of an event, which can be finished (perfective aspect) or ongoing (imperfective aspect; Comrie, 1976). In many languages, in particular tensed languages like French or English, tense is principally expressed by verb tenses through inflectional morphology (e.g., simple past, simple present, future, etc.) and temporal adverbials (e.g., yesterday, now, in 5 years, etc.). Grammatical aspect is also expressed by inflectional morphemes bound to verbs (e.g., walk*ed_perfective_* vs. was walk*ing_imperfective_*) and perfective or imperfective adverbials (e.g., last Monday vs. every Monday; Vetters, 1996; Grisot, 2018). Another linguistic device to convey aspect is lexical aspect, which refers to the temporal profile of the internal course of an event (Vetters, 1996; Grisot, 2018). It is related to verb semantics and to their argument structure, independent of marking for tense and grammatical aspect. For instance, following classification of Vendler (1957, 1967), verbs can be categorized as an activity, an achievement, an accomplishment, or a state depending on the use of the verb (e.g., “run” is an activity but “run a mile” is an accomplishment, depending on whether the final boundary of the situation—its end—is expressed or not; see Grisot, 2018 for more details about the differences between the four classes of lexical aspect in English and in French). Analyzing the use of morphological marking of tense and grammatical aspect on inflected verbs, classes of verbs (i.e., lexical aspect), and temporal adverbials in language production and comprehension could then be an effective way of assessing time processing abilities.

Interestingly, many studies focused on the processing of functional categories expressed by verbal morphology (such as tense, grammatical aspect, lexical aspect, and agreement) in AD. Using for instance sentence-completion tasks (Fyndanis et al., 2013, 2018; Manouilidou et al., 2020) or connected speech (e.g., Kavé and Levy, 2003; Sajjadi et al., 2012; see Auclair-Ouellet, 2015 for a systematic review) these studies aimed to specify the extent of morphological impairment in AD. Results revealed that all functional categories are impaired in AD participants, but not all to the same extent. Indeed, grammatical aspect is more impaired than tense and agreement (Fyndanis et al., 2013), and tense is more impaired than agreement (Fyndanis et al., 2013, 2018).

Psycholinguistic models of inflectional morphology generally explain deficits in terms of rule-processing impairments, phonological-encoding impairments, lexical-retrieval impairments, or reductions in cognitive resources (e.g., Ullman et al., 1997; Pinker, 1998; Faroqi-Shah and Thompson, 2004, 2007; Bastiaanse et al., 2011). However, studies of patients with post-stroke aphasia and primary progressive aphasia (PPA) suggest that those difficulties may be related to semantics, and more specifically to time conceptualization (e.g., Bates and Wulfeck, 1989; Auclair-Ouellet et al., 2016; Mack et al., 2021). Because it is known that semantic difficulties in AD become more severe with the progression of the disease, the question of whether time reference impairments may emerge as a result of time conceptualization impairments can be asked.

The relationship between time conceptualization and morphosyntactic expression of time is best understood through models of connected speech production. According to some models, building a temporal framework is essential to tell a coherent and complete story, but also to communicate efficiently. Telling a story requires the ability to recall an event and to create a mental representation of the situation described to communicate it verbally (Zwaan, 2008). Several dimensions are needed to create a complete event representation, including time (Zwaan et al., 1995a,b; Zwaan and Radvansky, 1998). When telling a story, the storyteller has to construct a temporal framework to ensure the listener understands the story. Because the temporal course of the story is not always continuous, as time shifts are quite common in narratives (Zwaan, 1996, 2008), the storyteller needs to go back and forth in time mentally to construct an event representation. Furthermore, to make sure that the listener will be correctly situated within the temporal framework and will understand the story, the storyteller also needs to give appropriate linguistic cues, such as verb tenses and temporal adverbials. As mentioned above, grammatical aspect and tense are more impaired than agreement in AD. Differences in the degree of impairment for different functional categories shed light on their underlying processes, as tense and grammatical aspect demand the integration of conceptual information in addition to grammatical information (Fyndanis et al., 2012). Likewise, the subjective and speaker-related dimensions involved in grammatical aspect processing (Fyndanis et al., 2012), which are absent for tense and agreement, may compromise temporality processing in AD. Since morphosyntactic marking of temporality relies on subjective dimensions associated with semantics and conceptual processes, it may be impaired in AD. These considerations raise the question of the extent to which mental representations of an event are integrated in memory (Zwaan, 2008) and then translated into language. In this perspective, time reference impairments may be related to time conceptualization impairments in AD.

In sum, time conceptualization impairments may manifest themselves as difficulties in mental time travel and in expressing temporality using lexical-semantic and morphosyntactic devices. However, the relationship between these two impairments in Mild Cognitive Impairment (MCI) and AD remains unclear. The goal of the present study is to perform a systematic review of the literature on the relationship between mental time travel impairments and time reference impairments in MCI and AD.

## Materials and Methods

This systematic review is based on the guidelines proposed by the Preferred Reporting Items for Systematic Reviews and Meta-Analysis (PRISMA; Page et al., 2021a). Using the Population-Intervention-Comparison-Outcomes (PICO) tool, the following research question was formulated: what influence does the ability of Alzheimer’s disease patients to conceptualize time have on their production and comprehension of time reference (tense and grammatical aspect *via* verbs and adverbs)?

This research question guided the choice of electronic databases, search strategy, study selection, and data extraction.

### Data Sources and Search Strategy

Three electronic databases were searched in February 2021: Ovid-MEDLINE (1946 to February 2021), Scopus (1960 to February 2021), and ProQuest-APA PsychInfo (1967-February 2021). We used keywords related to Alzheimer’s disease, time conceptualization, and time reference. Mesh-terms and truncators were used according to the particularities of each database. The detailed search strategy applied in the Ovid interface (MEDLINE) is available in Table 1. Following the new definition of Alzheimer’s disease of the NINCDS-ADRDA (McKhann et al., 2011), we included studies published in or after 2011.

**Table 1:** Search strategy applied *via* Ovid in Medline database.

Search strategy
1. Alzheimer disease/
2. Neurocognitive disorders/
3. mild cognitive impairment.ti,ab,kf.
4. time awareness.ti,ab,kf.
5. time perception.ti,ab,kf.
6. time course.ti,ab,kf.
7. event.ti,ab,kf.
8. temporal*.ti,ab,kf.
9. Memory, Episodic/
10. Mental Recall/
11. autobiographic* memory.ti,ab,kf.
12. (episodic adj3 thinking).ti,ab,kf.
13. (mental adj3 travel*).ti,ab,kf.
14. tense.ti,ab,kf.
15. verb inflection.ti,ab,kf.
16. narrati*.ti,ab,kf.
17. connected speech.ti,ab,kf.
18. discourse.ti,ab,kf.
19. discursi*.ti,ab,kf.
20. sentence.ti,ab,kf.
21. morpholog*.ti,ab,kf.
22. aspect*.ti,ab,kf.
23. time reference.ti,ab,kf.
24. morpheme.ti,ab,kf.
25. (temporal* adj2 adverb*).ti,ab,kf.
26. or/1–3
27. or/4–13
28. or/14–25
29. 26 and 27 and 28
30. limit 29 to yr = “2011-Current”

### Study Selection

Database searches were performed on February 11, 2021, and updated on November 8, 2021. The references were exported into reference management software (Zotero). One investigator (ES) removed all the duplicate records. The first screening step was then performed by two independent investigators (MS and ES). This screening was based on the title and abstract of each record yielded by the literature search. According to the eligibility criteria presented in Table 2, irrelevant studies were excluded. Then, after the initial screening, both investigators read the full text of the remaining articles. For the second step, the same criteria were used for the inclusion/exclusion of the articles.

**Table 2:** Overview of the eligibility criteria used for the selection of articles in the systematic review.

Eligibility criteria	Inclusion criteria	Exclusion criteria
*Population*	People with Alzheimer’s disease or with other neurodegenerative disorders leading to primary episodic deficits	Other neurodegenerative disorders without primary episodic memory deficits
*Intervention*	At least one task assessing time conceptualization and one task assessing time reference	Only tasks assessing time conceptualization or only tasks assessing time reference
*Publication type*	Peer-reviewed publication Written in English or French Original contributions to the literature Group or case study Publication year after 2011	(Systematic) reviews or meta-analysis studies Book chapters Conference reports
*Research parameters*	Time conceptualization measures (e.g., autobiographical interview, orientation, interval judgement, etc.) Language measures (time reference: e.g., number of inflected verbs, number of adverbials, sentence completion, etc.)	

During both steps of the selection process, disagreements on the inclusion of a record were resolved by discussion.

### Data Extraction

Relevant data were extracted by two independent reviewers (MS and ES) using a data extraction table. The following data were extracted: authors, year of publication, title, abstract, study goal, population, time conceptualization measures, language measures (time reference), other measures used, and results concerning temporality and language.

## Results

**Figure 1:**
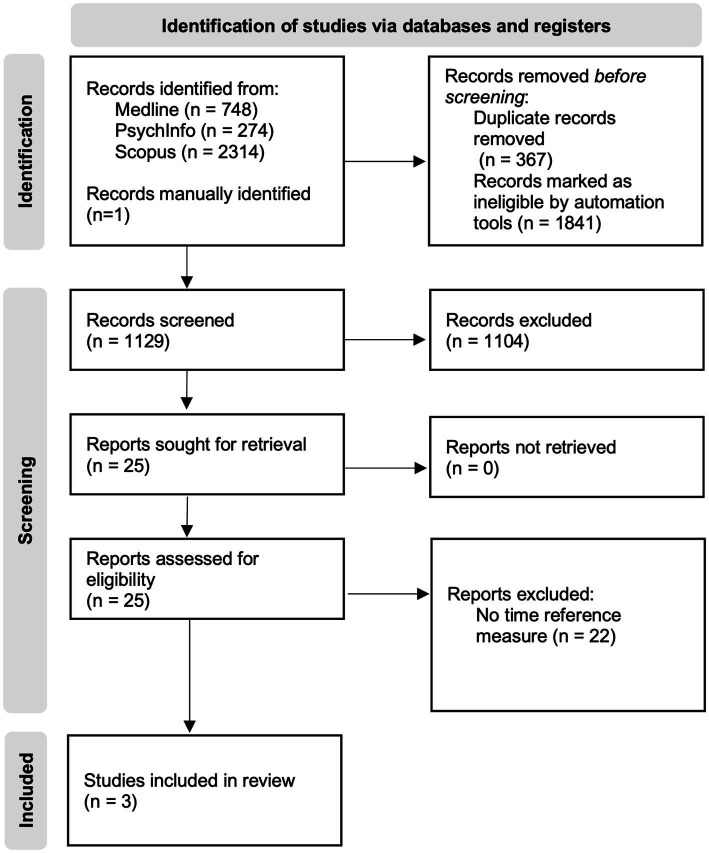
Flow diagram of the identification and selection of records, adapted from the Preferred Reporting Items for Systematic Reviews and Meta-Analysis (PRISMA) flow diagram (Page et al., 2021b).

The initial literature search in the three electronic databases yielded 3,209 records. The updated literature search performed in the same electronic databases resulted in 44 new records for Medline, 78 new records for Scopus, and five new records for PsychInfo, for a total of 3,336 records. Duplicates and records marked as ineligible by automation tools were removed, and 1,129 records remained. Screening of titles and abstracts resulted in 25 records. Two articles met the inclusion criteria after full-text reading. One additional study, which also met the inclusion criteria but was not identified by databases, was manually included. A flow diagram illustrating the identification and selection of the studies is provided in Figure 1.

### Characteristics of the Included Studies

Three articles were included in the systematic review. Ahmed et al. (2013), Irish et al. (2016), and Seixas-Lima et al. (2020) were the only studies reporting both time conceptualization measures and time reference measures.

Ahmed et al. (2013) studied connected speech of participants with AD at different stages of the disease (i.e., MCI, mild AD, and moderate AD). Their goal was to identify some language markers that could account for disease progression. To do so, they used a longitudinal design and used connected speech with a picture description task [i.e., Cookie Theft Picture from the Boston Diagnostic Aphasia Examination (Goodglass and Kaplan, 1972)] and neuropsychological tests [i.e., CAMCOG scores from CAMDEX; (Roth et al., 1986)]. Participants, who had no cognitive decline or MCI at the beginning of the study, were followed for 6–12 months until their deaths. Ahmed et al. (2013) showed changes in the CAMCOG scores and in the discourse of participants with AD as the disease evolved. More precisely, from a neuropsychological standpoint, their results revealed that scores on CAMCOG’s subtests of orientation, expression, remote, and recent memory significantly decreased with the progression of the disease. As far as language was concerned, the authors identified significant changes in semantic content, syntactic complexity (e.g., mean length of utterance, syntactic errors, and proportion of verbs with inflection), and lexical content (e.g., proportional frequencies of open class and closed class words) with the progression of the disease. They concluded that there was a progressive deficit in AD participants’ language, which could be detected by measures of semantic content, syntactic complexity, and lexical content. However, even if the authors reported both time conceptualization (i.e., scores on CAMCOG’s subtests of orientation, and remote and recent memory) and time reference measures (i.e., proportion of verbs with inflection), they did not analyze the potential link between them. Nevertheless, those two sets of measures appeared to progress at roughly the same rate and in the same direction over the course of the disease.

With a modified version of the Autobiographical Interview, Irish et al. (2016) analyzed how AD participants use the past and present tense in association with the production of internal details. They found that, in addition to producing fewer internal details, AD participants also used fewer inflected verbs to express past events (i.e., verbs produced in internal details) compared to control participants. In contrast, they used more inflected verbs than control participants for external details. Regarding tense usage, Irish et al. (2016) found no correlation between scores of episodic or autobiographical memory and the production of past tense verbs, but showed that AD participants produced an equivalent proportion of past and present verb tenses to express past events, while control participants used mostly the past tense, as expected considering the temporal framework of the event. According to the authors, this result can be explained by the tendency observed in AD to produce more external details (i.e., personal and general semantic details) in past events narratives. Indeed, because personal and general semantic information is not anchored in time, there is no need to use the past tense to express it. In this perspective, the predominant use of present tense may be a strategy used by AD participants to compensate their difficulties navigating through time, and recalling past events.

Seixas-Lima et al. (2020) also used a modified version of the Autobiographical Interview to investigate language impairments during episodic narratives by participants with amnestic MCI (aMCI), participants with the semantic variant of PPA (svPPA) and control participants. The authors expected to observe different patterns of language impairments according to the predominance of episodic or semantic memory difficulties. In addition to investigating the proportion of internal and external details in these populations, they also extracted different language measures (e.g., number of words, nouns, and verbs) from the narratives, as well as the proportion of inflected verbs produced (past and present tense inflections). Compared to the control group, aMCI participants produced significantly fewer internal details (i.e., episodic information), but more words in external details (i.e., semantic information). None of the other language measures differed between the two groups. On the contrary, the svPPA participants presented various language difficulties, especially when they expressed external details. The authors concluded that the difference between aMCI and controls was better explained by an episodic memory deficit rather than by language impairment.

Table 3 summarizes the principal characteristics of these three studies.

**Table 3:** Characteristics of the studies included in the systematic review.

Authors and date	Study’s goal	Population	Language measures (time reference)	Time conceptualization measures	Link between language and time
Ahmed et al., 2013	Identify language indicators of AD progression	9 patients with AD and 9 control participants	Connected Speech based on picture descriptions: proportion of verbs with inflection	CAMCOG^1^ scores (orientation, recent and remote memory)	No correlation analyses of language measures and time conceptualization measures
Irish et al., 2016	Investigate the incidence of the use of past tense in autobiographical narration	10 participants with svPPA, 10 participants with AD, and 10 control participants	Tense use: number of past and present tense verbs	Modified version of Autobiographical Interview: number of internal and external details	No correlation between scores of episodic or autobiographical memory and the production of past tense AD participants produce less internal details and less past tense compared to control participants
Seixas-Lima et al., 2020	Investigate whether participants with svPPA or aMCI show micro-linguistic impairments in internal or external details when producing an Autobiographical Interview	18 participants with svPPA +18 control participants 17 participants with aMCI +17 control participants	Inflection index: number of inflected verbs/number of verbs that could be inflected when adding a suffix Tense use: proportion of past and present tense inflections	Modified version of Autobiographical Interview: number of internal and external details	No correlation analyses of language measures and time conceptualization measures aMCI participants produce fewer internal details compared to control. No significant difference in tense usage were found between aMCI and control participants

1 *From CAMDEX (Roth et al., 1986)*.

*AD, Alzheimer’s disease; aMCI, amnesic mild cognitive impairment; and svPPA, semantic variant of primary progressive aphasia*.

## Discussion

The goal of this systematic review was to explore the potential link between the abilities in time conceptualization (e.g., mental time travel, episodic memory, and interval judgments), and in time reference (e.g., verbal inflection and temporal adverbials) in MCI and AD. Only three studies, met all selection criteria and were included in the review (Ahmed et al., 2013; Irish et al., 2016; Seixas-Lima et al., 2020). Ahmed et al. (2013) assessed time conceptualization in AD with subtests of the CAMCOG (Roth et al., 1986) focused on orientation, and remote and recent memory while Irish et al. (2016) and Seixas-Lima et al. (2020) used a modified version of the AI and analyzed the number of internal vs. external details. Concerning time reference measures, Ahmed et al. (2013) analyzed the proportion of verbs with inflection through connected speech with a picture description task, Irish et al. (2016) analyzed the number of present tense and past tense verbs produced by participants in their narratives, and Seixas-Lima et al. (2020) reported the proportion of past and present tense inflection. Even though both categories of measures of interest for our review were present in these three studies, only Irish et al. (2016) and Seixas-Lima et al. (2020) investigated the potential link between them. Interestingly, while Irish et al. (2016) found that AD participants produced fewer inflected verbs during the production of internal details compared to control participants, Seixas-Lima et al. (2020) found no difference in verbal inflection between aMCI and control participants. One reason for these different results in verbal inflection could be the nature of the different cognitive profiles in AD and aMCI participants. Indeed, in the study of Seixas-Lima et al. (2020), aMCI participants produced more external details (i.e., semantic information) compared to control participants, which may indicate that they still have difficulties to access episodic information and then to travel mentally in time. Although these difficulties are present, they may not yet be visible in verbal inflection (i.e., time reference), but may become so with the progression of the disease.

The results of this systematic review are quite surprising. Indeed, people with AD present well-documented time conceptualization difficulties as well as time reference difficulties, whose functional origins are widely debated (e.g., Fyndanis et al., 2013, 2018). Yet, very little work made connections between these two facets of time processing. We believe the likely reasons behind this gap in the literature are two-fold: firstly, the existence of separate lines of inquiries and research fields, and second, the emphasis on the dissociations between regular and irregular verbs in studies of morphology.

### Different Research Fields

Our systematic review shows that only two studies focused on the potential relationship between how people with AD conceptualize time and express it in language. This result suggests that this relationship has not been much studied as yet, or at least not in the way we conceptualized it. One possible explanation is that our selection criteria were based on two types of literature having both different methods, theoretical backgrounds, and objectives: studies on time conceptualization and mental time travel on the one hand (e.g., Addis and Tippett, 2004; Addis et al., 2009; Irish et al., 2011, 2012a,b), and studies on verbal inflectional morphology on the other hand (e.g., Kavé and Levy, 2003; Sajjadi et al., 2012; Fyndanis et al., 2013, 2018; Manouilidou et al., 2020).

Studies focusing on time conceptualization and mental time travel in AD rarely integrate verbal inflection measures. This could be due to the idea, which has long been predominant, that the morphosyntactic abilities of people with AD are relatively spared, at least in the early stage of the disease. Actually, it is well-known that morphosyntax is not always spared in AD (see Auclair-Ouellet, 2015 for a systematic review). For instance, some studies showed that people with AD have difficulties with verbal inflection (e.g., Cuetos et al., 2007; Sajjadi et al., 2012; Fyndanis et al., 2018) and produce shorter and less complex sentences (e.g., de Lira et al., 2010; Sajjadi et al., 2012; Boschi et al., 2017).

On the other hand, when focusing on verbal inflection, studies rarely explore the conceptual level of time representation and expression. Some authors like Fyndanis et al. (2013, 2018) discussed, in passing, the question of a potential link between time conceptualization and time reference in AD to explain some of their results. However, the notion of “conceptual level” as it is included for example in model of language production of Levelt (1989, 1999), is not clearly defined in this context. It seems that Fyndanis et al. (2013, 2018) link the implication of the conceptual level mostly to the number of cognitive resources (e.g., working memory) needed to inflect verbs. While they consider that the conceptual level could impact verbal inflection in AD, they do not report conceptual measures (i.e., mental time travel, episodic memory, or time judgment measures) to test this hypothesis.

### Focus on Verb Regularity

Over the last 25 years, many theories have been proposed to account for morphosyntactic difficulties in acquired language disorders. The reasons put forward to explain those difficulties included impairments of rule processing or lexical retrieval (Pinker, 1998), which would be related to impairments of procedural and declarative memory, respectively (Ullman et al., 1997). Because the double dissociations in regular and irregular verb impairment put forward by these models were not systematically observed (e.g., Faroqi-Shah and Thompson, 2004, 2007; Faroqi-Shah, 2007; Macoir et al., 2013; Auclair-Ouellet et al., 2016, 2018), other models suggested that the difficulties were related to phonological or semantic processing impairments (e.g., Joanisse and Seidenberg, 1999; McClelland and Patterson, 2002; Faroqi-Shah and Thompson, 2004, 2007; Bastiaanse et al., 2011). Regarding the latter explanation, it was proposed to explain difficulties mostly when producing the past tense of irregular verbs in English, but a few studies considered a more conceptual contribution to morphology. Faroqi-Shah and Thompson (2004, 2007) proposed the *Diacritical Encoding and Retrieval hypothesis* (DER). For these authors, it is the activation from the conceptual level or the guidance of diacritical features (e.g., tense, grammatical aspect, person, or mood) to select the appropriate inflectional morpheme that could cause verbal inflection impairments. Focusing on the tense feature, Bastiaanse et al. (2011) proposed the *PAst DIscourse LInking Hypothesis* (PADILIH). According to them, the past tense requires more cognitive resources than the present and the future tense because it is linked with the discourse. Indeed, since past events do not occur at the same time as the speech time, they need to be linked. This link is not mandatory for present events as the event and its speech time are simultaneous. They consider the future as a specific case of the present.

All these theories are of great interest to the field, but they are still insufficient to explain difficulties encountered by people with acquired language disorders. Furthermore, conceptual processes have not yet been seriously considered as playing an essential role in verbal inflection. While Faroqi-Shah and Thompson (2004, 2007) proposed that activation of diacritical features (i.e., conceptual process) could be the origin of verbal inflection impairments, they did not explain how the activation of diacritical features could be impaired in the inflectional process. Currently, no consensus concerning the origin of inflection impairment has emerged from this research. Even though the role of conceptual processes has been discussed in research on inflectional morphology in post-stroke aphasia (Bates and Wulfeck, 1989) and primary progressive aphasia (Auclair-Ouellet et al., 2016; Mack et al., 2021) it is still marginally considered as a potential origin of inflection difficulties. This also seems to apply to research on AD; as for instance, Ahmed et al. (2013) did not explore the link between the scores of their AD participants in orientation, remote and recent memory and their scores in verbal inflection, even if these two domains of performances decreased significantly with the progression of the disease.

Our results show that the integration of the conceptual level in psycholinguistic studies is still difficult, but also that studies focusing on time conceptualization do not consider linguistic processes as potential indicators of how people conceptualize time, with the exception of the studies of Irish et al. (2016), and Seixas-Lima et al. (2020). These authors suggest that an interaction between language and memory (i.e., semantic or episodic) may impact language production, and that language impairment could emerge as the result of a memory deficit. More precisely, Irish et al. (2016) proposed that progressive episodic memory impairment in AD could be related to the pattern of verb tense usage in narration of past events. They hypothesized that the difficulty for AD participants to mentally navigate through time, and especially to retrieve past events, could be directly related to the decrease in the use of the past tense. Since the present tense is not aligned with the temporal framework of the recall event, the higher proportion of present tense verbs in AD participants’ narrations could signal the use of a more accessible tense as a compensatory strategy. However, they did not find correlations between scores of episodic or autobiographical memory and the production of past tense in AD participants. They attributed those results to their small sample size of AD participants and floor effects of this group on memory tasks. The authors concluded by highlighting the importance of further studies with bigger samples to explore this link. Surprisingly, it seems that this has yet to be done. In the following section, we propose additional considerations for future studies.

### Time Conceptualization Deficits and Language

Impairments in time conceptualization have been linked with other aspects of language. For example, Pistono et al. (2016) studied the usage of pauses during autobiographical discourse in AD and showed that AD participants’ discourse contained more between-utterance pauses than control participants’ discourse. AD participants’ pauses were also longer compared to those of control participants. For the authors, the difference between AD participants and controls indicates that AD participants need more time to remember past events and to plan their autobiographical discourse. It suggests that mental time travel impairment in AD impacts the way past events are translated into discourse.

Research has shown that mental time travel deficits can be measured in AD through tense usage in discourse (e.g., Irish et al., 2016), and pause usage (e.g., Pistono et al., 2016). These results raise questions about other potential language indicators of mental time travel difficulties. Indeed, language is the main way of expressing concepts of time (i.e., past, present, or future) and these concepts are an essential part of event narration. As time is principally marked in language *via* verbal inflectional morphology and temporal adverbials, it seems important to pursue the investigation of a potential link between how people with AD conceptualize time and how they translate it in language. In this perspective, future studies should integrate time conceptualization measures as well as time reference measures to further explore this potential link.

## Conclusion

Each of our actions is temporally anchored and represented on the mental timeline. While autobiographical memory and mental time travel enable us to relive past events or to imagine future ones, language is an important way of conveying experiences and observing mental time travel processes in others. The number of internal and external details in narrations and time reference (verbal inflection and temporal adverbs use) are two language indicators of mental time travel which can both be impaired in AD. Even if mental time travel and time reference difficulties could be related in AD, the literature published so far is not conclusive. Considering the significant overlaps in the understanding of temporality as it is experienced in mental time travel and expressed in language, studies investigating the role of time conceptualization in time reference in people with AD are warranted.

## Data Availability Statement

The raw data supporting the conclusions of this article will be made available by the authors, without undue reservation.

## Author Contributions

ES, NA-O, and MF contributed to conception and design of the study. ES organized the database. ES and MS analyzed the data. ES wrote the first draft of the manuscript. MS, CG, and NA-O wrote sections of the manuscript. All authors contributed to the article and approved the submitted version.

## Funding

This work was supported by the Swiss National Science Foundation (grant number 10001F_197862 attributed to MF).

## Conflict of Interest

The authors declare that the research was conducted in the absence of any commercial or financial relationships that could be construed as a potential conflict of interest.

## Publisher’s Note

All claims expressed in this article are solely those of the authors and do not necessarily represent those of their affiliated organizations, or those of the publisher, the editors and the reviewers. Any product that may be evaluated in this article, or claim that may be made by its manufacturer, is not guaranteed or endorsed by the publisher.
